# Sonographic Phrenic Nerve Changes in Amyotrophic Lateral Sclerosis

**DOI:** 10.3390/medicina59101745

**Published:** 2023-09-29

**Authors:** Ovidijus Laucius, Justinas Drūteika, Renata Balnytė, Kęstutis Petrikonis, Miglė Ališauskienė, Antanas Vaitkus

**Affiliations:** Department of Neurology, Medical Academy, Lithuanian University of Health Sciences, 44307 Kaunas, Lithuania

**Keywords:** phrenic, nerve, sonography, structural changes

## Abstract

Amyotrophic lateral sclerosis (ALS) is a progressive neurodegenerative disease that affects both the upper and lower motor neurons in the nervous system, causing muscle weakness and severe disability. The progressive course of the disease reduces the functional capacity of the affected patients, limits daily activities, and leads to complete dependence on caregivers, ultimately resulting in a fatal outcome. Respiratory dysfunction mostly occurs later in the disease and is associated with a worse prognosis. Forty-six participants were included in our study, with 23 patients in the ALS group and 23 individuals in the control group. The ultrasound examination of the phrenic nerve (PN) was performed by two authors using a high-resolution “Philips EPIQ 7” ultrasound machine with a linear 4–18 MHz transducer. Our study revealed that the phrenic nerve is significantly smaller on both sides in ALS patients compared to the control group (*p* < 0.001). Only one significant study on PN ultrasound in ALS, conducted in Japan, also showed significant results (*p* < 0.00001). These small studies are particularly promising, as they suggest that ultrasound findings could serve as an additional diagnostic tool for ALS.

## 1. Introduction

Amyotrophic lateral sclerosis (ALS) is a progressive neurodegenerative disease that affects upper and lower motor neurons, resulting in muscle weakness, severe disability, and ultimately, a fatal outcome [1,2,3]. The cause of ALS remains unknown for most individuals affected by the disease. Sporadic cases account for 90–95% of all cases, while genetic factors are confirmed in 5–10% [4,5]. Recent data shows that the incidence varies from 0.6 to 3.8 cases per 100,000 population, with a global incidence of approximately 1.6–1.76 cases per 100,000 population [5,6,7,8]. The main ALS center in Lithuania, located in the Hospital of the Lithuanian University of Health Sciences Kaunas Clinics, identifies approximately 20 new cases per year. ALS can occur at any age, but the incidence increases with each decade, particularly after the age of 40. The disease is most commonly diagnosed in older patients, with a peak age at the time of diagnosis of 51–66 years [9,10,11]. In all age groups, there is a higher occurrence of ALS in men compared to women. The peak in men is 85–89 years, while for women, it is 80–84 years. Multiple pathophysiological mechanisms are believed to contribute to motor neuron degeneration [12]. Due to various pathogenic factors, ALS presents with a wide range of clinical features and symptoms, making the diagnosis complex and challenging [13]. The diagnosis of ALS is based on clinical criteria and monitoring the progression of the disease. Additional tests can help avoid diagnostic errors and provide further insights into the disease. While respiratory muscle weakness is only observed as the initial symptom in a small number of ALS patients [14], respiratory dysfunction occurs later in the disease course and is associated with a poorer prognosis [15]. Gradual loss of phrenic nerve (PN) function leads to weakness, paralysis, and ultimately the death of the patient [16]. Studies using ultrasound and PN conduction can aid in assessing respiratory function and predicting patient survival [17,18,19,20].

Despite the advancements in innovative technologies and the current diagnostic capabilities in laboratories and instrumental methods, the existing diagnostic methods for amyotrophic lateral sclerosis (ALS) do not always ensure early detection, given the complexity of the diagnosis process resulting from a heterogeneous range of clinical symptoms. These factors drive the search for new and simpler diagnostic methods.

We raised the hypothesis that motor nerve size reduction in ultrasound could be one of the first signs in the diagnosis of ALS. There is still limited data and debate regarding which nerves are the most specific and sensitive to be examined by ultrasound. We hope that, due to the affected respiratory function, the PN might be the first damaged nerve in ALS disease. PN ultrasound and conduction studies provide hope that structural changes observed through PN ultrasound may become a diagnostic tool for determining ALS disease and evaluating respiratory function. This could enable more effective patient monitoring and care. 

We hypothesize that changes of phrenic nerve in terms of cross-sectional area (CSA), as well as alterations in nerve structure changes such as heterogeneity and in echogenicity, could potentially offer valuable insights and enable early diagnosis and prognosis assessment of Amyotrophic Lateral Sclerosis (ALS).

## 2. Materials and Methods

### Study Subjects and Ethical Statement

The study was conducted in accordance with the principles of the Declaration of Helsinki, and informed consent was obtained from all participants. The study protocol was approved by the Kaunas Regional Biomedical Research Ethics Committee, with bioethical permission No. BE-2–40, issued on 17 May 2022. An analysis of scientific literature, medical documentation of participants, as well as laboratory and instrumental research data, was performed.

All participants were enrolled, and the study was conducted at the Department of Neurology of the Lithuanian University of Health Sciences. The participants were divided into two groups: Group 1 consisted of patients with ALS (N = 23), who all met the “Gold Coast” criteria (Shefner et al. 2020). Group 2 served as the control group (N = 23). Each participant underwent an interview and filled out questionnaires to collect clinical and demographic data, including age, gender, height, body mass index (BMI), hip and waist circumference, duration of illness, and ALS-FRS-R score. All participants underwent a PN ultrasound examination. In the control group, patients who matched the age and gender of the ALS patients were invited to participate. The cross-sectional area (CSA) values of the PN in the control group were determined by measuring personal staff members and patients who were over 18 years old. Participants who had signs and symptoms of other neurodegenerative diseases, polyneuropathies, neuromuscular junction disorders, endocrine disorders, oncological diseases, or other concomitant diseases that could affect respiratory function, as well as those taking medications that could affect respiration, were excluded from the study. Both groups were matched by age, gender, height, weight, hip and waist circumference, and BMI.

## 3. Ultrasound Examination

**Figure 1:**
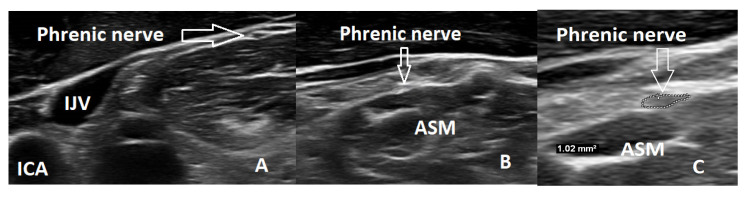
(**A**) Normal phrenic nerve ultrasound in a healthy volunteer. (**B**) Phrenic nerve ultrasound in an ALS participant. (**C**) Methodology for measuring the cross-sectional area of the phrenic nerve. ASM—anterior scalene muscle. IJV—internal jugular vein. ICA—internal carotid artery.

The ultrasound examination of the phrenic nerve was performed by two authors using a high-resolution “Philips EPIQ 7” ultrasound machine with a linear 4–18 MHz transducer (CE 0086). The transducer was placed in a transverse plane above the level of the clavicle in the neck region, at the level of the levator scapulae muscle, where the nerve attaches to the anterior scalene muscle and the neck muscles. In B-mode ultrasound images, a spindle-shaped structure with a hypoechoic appearance and a more hyperechoic periphery was visualized in the connective tissue sheath on the anterior surface of the scalene muscle, which was identified as the PN (Figure 1). To ensure accurate differentiation of the nerve from vascular structures, a Doppler ultrasound mode was used during the examination. During the examination, the quantitative and qualitative characteristics of the PN were evaluated. The texture of the nerve was assessed based on homogeneity (homogeneous or heterogeneous) and echogenicity (hypoechoic, isoechoic, or hyperechoic). The cross-sectional area of the PN was measured in a transverse plane by tracing the hypoechogenic nerve area at the border of hyperechogenicity using the methodology described by Walter et al. (2019). The cross-sectional area was measured by two examiners. Each observer measured one side three times with a 0.01 mm^2^ error margin, and the average of the three measurements was calculated. Then, the grand average of each side was determined by the observers. Both examiners were blinded to each other’s examination results.

## 4. Statistical Analysis

The data analysis involved the use of both descriptive and comparative statistical analysis methods, namely Microsoft Excel and the IBM SPSS v29 as the statistical software used in the study Quantitative values within the sample were presented using median, minimum, and maximum values, as well as the mean of the PN area with the standard deviation. Non-parametric tests, such as the Mann–Whitney U test, were used to compare values between groups. Differences in data were analyzed using the Kruskal–Wallis test. Statistical hypotheses were tested, and significant differences and dependencies were defined as *p* < 0.05.

## 5. Results

A total of 46 participants were included in the study, with 23 patients in the ALS group and 23 individuals in the control group. In the ALS group, there were 9 women (39.1%) and 14 men (60.9%). The median age of the ALS group on the day of the PN ultrasound exam was 57 years (range: 31–70). Among the women in the ALS group, the median age was 59 years (range: 31–70), and among the men, the median age was 57 years (range: 46–70). The control group consisted of 11 women (47.8%) and 12 men (52.2%), with a median age of 58 years (range: 46–65) for the entire group. There were no significant differences in age, gender, or anthropometric measures between the study groups (*p* > 0.05). With the Kruskal–Wallis test used, no statistically significant differences were observed between the study groups for any of the variables (*p* > 0.05). The demographic and clinical characteristics of the study groups are presented in Table 1.

Participants in the ALS group were categorized into four different groups based on the clinical form in which the disease initially presented. The distribution of clinical forms and sex in each group is displayed in Table 2. Additionally, the duration of illness and ALS-FSR-R scores by gender were included in the analysis.

**Figure 2:**
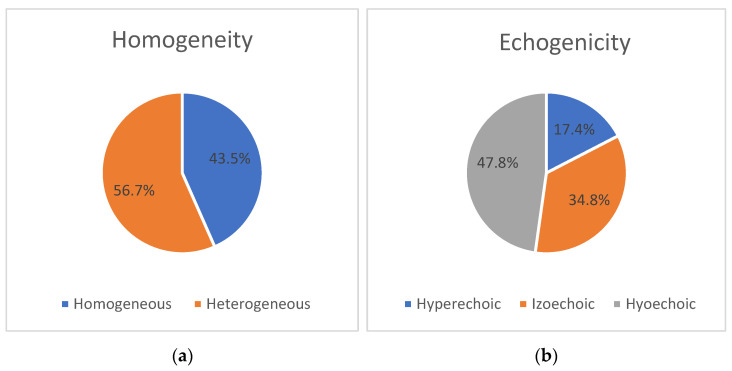
Ultrasonic features of the right PN nerve in the group of patients diagnosed with amyotrophic lateral sclerosis (ALS) (n = 23), showing homogeneity (**a**) and echogenicity (**b**). Abbreviations: ALS—amyotrophic lateral sclerosis; %—percentage.

**Figure 3:**
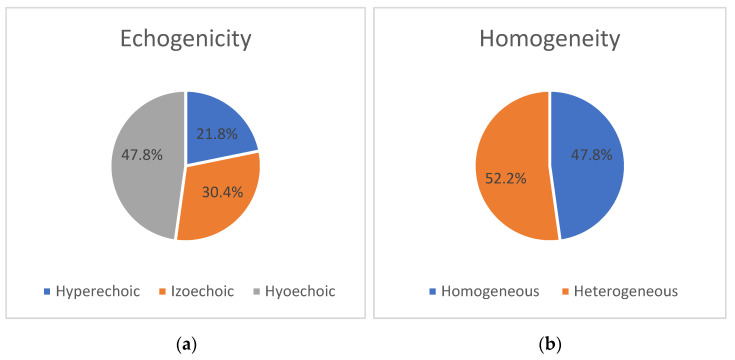
Ultrasonic features (homogeneity (**a**) and echogenicity (**b**)) of the left PN in the group of patients with amyotrophic lateral sclerosis (ALS) (n = 23). Abbreviations: ALS—amyotrophic lateral sclerosis; %—percentage.

During the ultrasound examination of the PN, heterogeneity was observed on both sides (43.5% on the right and 52.2% on the left). Among the patients, nerve heterogeneity was present on at least one side in 14 individuals (60.9%). In the remaining cases, the nerve showed a homogeneous appearance without any interruptions (13 (56.5%) on the right and 11 (47.8%) on the left). Changes in echogenicity of the PN were detected bilaterally, but there were no significant differences in nerve echogenicity between sides. The nerve was found to be isoechoic in eight patients (34.8%) on the right and seven patients (30.4%) on the left, hyperechoic in four patients (17.4%) on the right and five patients (21.8%) on the left, and hypoechoic in 11 patients (47.8%) on both sides (*p* > 0.05). The distribution of ultrasonic features, including homogeneity and echogenicity, of the right and left PNs in the ALS group is presented as a percentage in Figure 2 and Figure 3.

All participants in the control group exhibited homogeneous structures in both the right and left PNs. Among individuals in the control group, hypoechogenic structures were more prevalent (69.6% on the right, 78.3% on the left), while isoechoic structures were less common (30.4% on the right, 21.7% on the left), and no nerves with hyperechogenic structures were observed.

Figure 2 and Figure 3 show the distribution of ultrasonographic features (homogeneity and echogenicity) of the right and left phrenic nerves in the ALS group.

In the control group, the median cross-sectional area of the PN was 1.16 mm^2^ (range: 0.63–1.36) on the right side and 1.09 mm^2^ (range: 0.63–1.20) on the left side. However, in patients with ALS, it was observed that the cross-sectional area of the PN was significantly smaller on both the right (*p* < 0.001) and left (*p* < 0.001) sides compared to the control group. Detailed measurements are shown in Table 3.

## 6. Discussion

Despite the advancements in innovative technologies and the current diagnostic capabilities in laboratories and instrumental methods, the existing diagnostic methods for amyotrophic lateral sclerosis (ALS) do not always ensure early detection, given the complexity of the diagnosis process resulting from a heterogeneous range of clinical symptoms. These factors drive the search for new and simpler diagnostic methods. In recent decades, the hypothesis of nerve size reduction due to ongoing axonal degeneration has sparked interest in the use of peripheral nerve ultrasound for ALS diagnosis and disease monitoring [21]. As respiratory system impairment is inevitable due to motor neuron loss in ALS, previous studies have investigated PN conductivity, diaphragm radiology, and respiratory functionality to understand the role of the PN in ALS [20,22,23,24,25,26,27,28,29].

Our study revealed that the PN is significantly smaller on both sides in ALS patients compared to individuals without the disease (*p* < 0.001). Only one significant study on PN ultrasound in ALS, conducted in Japan, also showed significant results (*p* < 0.00001).

Comparing this study with our own, we noticed that Surat and colleagues recorded larger PN cross-sectional areas in both the control and ALS patient groups. Upon analyzing the Japanese study, we concluded that these differences could be attributed to variations in different PN evaluation sites, ultrasound machine and probe technical differences, and different researchers involved. We also evaluated and compared the ultrasound parameters of the PN in ALS patients with individuals in a control group who were matched in age and gender. There were no significant differences in age, gender, height, waist or hip circumference, weight, or BMI between the two groups. The median age of the ALS group corresponded to the age peak of the disease reported in the scientific literature [9,10,11].

In our study, there was no significant difference in the cross-sectional areas of the PN (PN) between the right and left sides in ALS patients. This finding is consistent with our own study results, which showed a median cross-sectional area of 0.81 mm^2^ (range: 0.58–1.07 mm^2^) on the right side and 0.81 mm^2^ (range: 0.54–0.95 mm^2^) on the left side. The observed changes in PN cross-sectional area between the study groups, as reported by us and Surat et al., are supported by postmortem studies that document significant loss of large myelinated axons, predominantly in distal regions, and significant distal axonal PN atrophy [30]. Oxidative damage, which is implicated in the pathogenesis of ALS, contributes to axonal dysfunction and degradation [31]. Oxidative stress, which affects diffuse cellular processes, can explain the diffuse degeneration of both the right and left PNs. Therefore, we believe that significantly reduced PN size in ALS patients may be one of the indicators of ongoing neurodegeneration.

There is limited data on PN sonography in the literature [32,33,34,35,36]. These studies primarily focus on the nerve’s anatomical features and the quantitative analysis of its diameter or cross-sectional area. Surat et al. also measured a larger cross-sectional area of the right PN (*p* < 0.01) [37]. In another small ultrasound study, Canella et al. [32] also found a larger CSA of the right PN, although the difference between the sides was not significant. To ensure accuracy and avoid errors due to the varying experience of researchers, our ultrasound study was conducted with two independent researchers to obtain the most reliable results. In the control group, we found that the right PN was significantly larger than the left PN 1.16; (range: 0.63–1.36) mm^2^ and 1.09 (range: 0.63–1.20) mm^2^, respectively; *p* < 0.05).

Although the nerve appears as a hypoechoic structure with a hyper-echoic border peripherally on ultrasound, qualitative parameters such as homogeneity and echogenicity are not thoroughly evaluated in studies, making it unclear what the sensitivity and specificity of PN sonography are. In our study, we found a significant difference in homogeneity between the study groups (*p* < 0.001). However, the results are questionable, as no heterogenic nerve structures were detected in the control group during the ultrasound examination. Doubts about the results of this study are reinforced by the knowledge of morphological changes observed in peripheral and cranial nerves in healthy individuals due to age-related natural nerve degeneration [38].

We also did not find a significant difference in nerve echogenicity (*p* > 0.05), which may be attributed to the small size of our study sample. These findings suggest that the observed morphological changes are non-specific to the degeneration that occurs with ALS and are not directly related to the reduction of the phrenic nerve’s cross-sectional area. Therefore, large-scale studies are necessary to obtain more accurate assessments and draw significant conclusions.

One of the objectives of this study was to identify the relationship between ultrasound changes in the PN and clinical characteristics of ALS, such as the form of the disease, duration, and functional status. However, we did not find any significant correlations (*p* > 0.05). Similarly, a study conducted by Surat et al. [37] also found no significant correlation between decreased PN cross-sectional area and any clinical variables, including disease duration, onset type, and ALS-FRS-R functional score). The authors suggest that the lack of correlation between reduced PN CSA and disease duration may indicate changes in PN morphology in the early stages of the disease. This is consistent with evidence of distal axonal degeneration occurring before the manifestation of symptoms in animal models of motor neuron disease.

Although the evaluation of other peripheral nerves showed very small long-term nerve changes [39], researchers acknowledge that a relatively small change in nerve cross-sectional area requires a large sample size to obtain significant results and draw conclusions about the potential of ultrasound examination. However, these studies suggest that ultrasound measurements could become crucial biomarkers in the future. Due to the limited literature data and the small sample size of our ALS patient group, we cannot provide clear and definite conclusions.

This is the first study of its kind in Lithuania to attempt to link ultrasound-observed changes in the PN to its dysfunction in patients with ALS. Therefore, future studies should aim to more thoroughly evaluate the relationship between these tests and ultrasound-observed changes in PN in our study. As mentioned earlier, the limited sample size is one of the shortcomings of this study that may have affected the reported results.

## 7. Conclusions

In this context, the results of this small study are particularly promising, as they suggest that sonographic findings could serve as a diagnostic tool for ALS. However, it is important to note that further research is necessary to validate these results and determine the full extent of their diagnostic utility. Continued investigation in this area will help refine the methodology, improve accuracy, and establish the reliability of sonographic techniques for ALS diagnosis. Such advancements can lead to a better understanding of the pathophysiology of ALS and potentially develop more effective treatments for this debilitating disease.

## Figures and Tables

**Table 1:** Demographic and clinical characteristics of the study groups.

Variable	Control Group	ALS Group
Number	23	23
Age years ± min/max (range, years)	58 (46–65)	57 (31–70)
Sex ± min/max (M:F)	11:12	9:12
Weight cm ± min/max (range, Kg)	75 (49–124)	75 (48–102)
Height cm ± min/max (range, cm)	170 (155–190)	170 (157–189)
BMI Kg/m^2^ ± min/max (range, Kg/m^2^)	25.32 (18.67–36.63)	25.35 (18.99–36.57)
Hip circumference cm ± min/max (range, cm)	85 (92–123)	87 (79–121)
Waist circumference cm ± min/max (range, cm)	101 (71–125)	101 (60–115)

ALS—amyotrophic lateral sclerosis. BMI—body mass index.

**Table 2:** ALS group distribution by sex and forms.

Variable	ALS group		
Sex and Number	Female (N-9)	Male (N-14)	All ALS (N-23)
UMN, % of form LMN, % of form Bulbar/pseudobulbar, % of form Composite, % of form	1 (33%) 3 (33%) 3 (43%) 2 (50%)	2 (67%) 6 (67%) 4 (57%) 2 (50%)	3 (13%) 9 (39%) 7 (31%) 4 (17%)
Duration of illness, months ± min/max	12 (6–30)	12 (5–44)	12 (5–44)
ALS-FRS-R, score ± min/max	35 (32–46)	40 (24–44)	39 (24–46)

N—number of participants; ALS—amyotrophic lateral sclerosis; %—percentage; UMN—upper motor neuron; LMN—lower motor neuron. ALS-FRS-R—revised version of the functional status assessment scale for patients with amyotrophic lateral sclerosis.

**Table 3:** Cross-sectional area of the PN nerve in both groups.

Side of PN	Control Group	ALS Group	*p* Value
PN right mm^2^ ± min/max (range, mm^2^)	1.16 (0.63–1.36)	0.81 (0.58–1,07)	<0.001
PN left mm^2^ ± min/max (range, mm^2^)	1.09 (0.63–1.20)	0.81 (0.54–0.95)	<0.001

Abbreviations: ALS—amyotrophic lateral sclerosis; PN—phrenic nerve; mm^2^—square millimeters; *p*-value—level of significance.

## Data Availability

Data is unavailable due to privacy or ethical restrictions.
